# Knocking out Ornithine Decarboxylase Antizyme 1 (*OAZ1*) Improves Recombinant Protein Expression in the HEK293 Cell Line

**DOI:** 10.3390/medsci6020048

**Published:** 2018-06-08

**Authors:** Laura Abaandou, Joseph Shiloach

**Affiliations:** 1 Biotechnology Core Laboratory, National Institute of Diabetes and Digestive and Kidney Diseases, National Institutes of Health, Bethesda, MD 20892, USA; laura.abaandou@nih.gov; 2 Department of Chemistry and Biochemistry, George Mason University, Fairfax, VA 22030, USA

**Keywords:** protein expression, antizyme 1, ornithine decarboxylase, CRISPR, human embryonic kidney 293 (HEK293)

## Abstract

Creating efficient cell lines is a priority for the biopharmaceutical industry, which produces biologicals for various uses. A recent approach to achieving this goal is the use of non-coding RNAs, microRNA (miRNA) and small interfering RNA (siRNA), to identify key genes that can potentially improve production or growth. The ornithine decarboxylase antizyme 1 (*OAZ1*) gene, a negative regulator of polyamine biosynthesis, was identified in a genome-wide siRNA screen as a potential engineering target, because its knock down by siRNA increased recombinant protein expression from human embryonic kidney 293 (HEK293) cells by two-fold. To investigate this further, the *OAZ1* gene in HEK293 cells was knocked out using CRISPR genome editing. The *OAZ1* knockout cell lines displayed up to four-fold higher expression of both stably and transiently expressed proteins, with comparable growth and metabolic activity to the parental cell line; and an approximately three-fold increase in intracellular polyamine content. The results indicate that genetic inactivation of *OAZ1* in HEK293 cells is an effective strategy to improve recombinant protein expression in HEK293 cells.

## 1. Introduction

The production of recombinant proteins from mammalian cells for therapeutic and research purposes has always been associated with the challenge of improving production. As a result, volumetric productivity has been increased significantly in the last two decades by media optimization, careful process control [1,2,3], genetic modification [4,5,6], and cell line selection [1]. Chinese hamster ovary (CHO) cells are currently the main industrial producers of recombinant therapeutic proteins [7,8] because of their efficient growth and high expression characteristics. These and other non-human producers, such as baby hamster kidney (BHK21) cells and murine myeloma cells (NS0 and Sp2/0) [9], are limited by their ability to correctly perform post-translational modifications, which are important for proper activity of the produced protein [10]. Therefore, efforts are ongoing to improve growth and expression of recombinant protein from human cell lines, such as human embryonic kidney 293 (HEK293) and fibrosarcoma HT-1080 [11]. Recent research conducted by Su et al. looked at the possibility of improving protein expression from HEK293 cells by utilizing non-coding RNA, such as microRNA (miRNA) and small interfering RNA (siRNA) [12,13,14]. The study was conducted in two directions; first, the focus was on finding microRNA that have a direct effect on protein expression; and the second was focused on finding specific genes whose inactivation by siRNA improved expression. This work was performed by implementing two independent high throughput screenings of the non-coding RNAs. By screening the effect of 23,000 different siRNAs, ten genes whose knock-down triggered higher expression of recombinant luciferase were selected for further studies. Among the ten identified genes, ornithine decarboxylase antizyme (*OAZ1*) attracted special attention, because its inhibition improved the expression of luciferase with minimal effects on cell growth and viability. Additionally, the role of *OAZ1* in polyamine metabolism has been well studied [15,16], making it a promising target to explore the connection between *OAZ1* inhibition and the level of different polyamines. The *OAZ1* protein negatively regulates polyamine biosynthesis by degradation of ornithine decarboxylase. This enzyme catalyzes the decarboxylation of ornithine to form putrescine, a committed step in polyamine biosynthesis. This connection was investigated [17], and preliminary information showed that transient inhibition of *OAZ1* in HEK293 cells expressing luciferase by siRNA was associated with increased luciferase expression and a higher intracellular concentration of putrescine. Creating a permanent cell line lacking *OAZ1* should, therefore, be the next step in evaluating the potential of the *OAZ1* knockout cell line as an efficient producer of recombinant proteins from HEK293, and perhaps other mammalian cell lines.

Several gene editing tools are currently available, such as transcription activator-like effector nuclease (TALENS), zinc finger nucleases (ZFNs), and the clustered regularly interspaced short palindromic repeats (CRISPR)/Cas system, which consists of a Cas endonuclease directed to cleave a target sequence by a guide RNA. The CRISPR system, with its unprecedented level of simplicity, efficiency, and ability to carry out multiplexed mutations [18], was chosen to create an *OAZ1* deleted cell line. In this report, we evaluate the growth and production capabilities of the created cell line for production of recombinant proteins in both stable and transient transfection systems.

## 2. Materials and Methods

**Cell line:** HEK293 stably transfected with the luciferase gene of *Photinus pyralis* under a cytomegalovirus (CMV) promoter (CMV-Luc2-Hygro HEK293, Promega ID# CAS140901, Madison, WI, USA). This cell line will be referred to as the parental cell line in the text.

**Cell culture:** Cells were grown in adherent cultures in Dulbecco’s Modified Eagle Medium (DMEM Gibco cat# 11995–040, Grand Island, NY, USA) supplemented with 10% fetal bovine serum (FBS, Atlanta Biologicals cat# S11150H, Flowery Branch, GA, USA), 100 units/mL of penicillin, and 100 µg/mL of streptomycin (Gibco cat# 15140–122, Grand Island, NY, USA). The cultures were incubated in a humidity-controlled incubator at 37 °C and 5% CO_2_.

### 2.1. Construction of CRISPR/Cas9 Lentiviral Particles with Single Guide RNAs Targeting the OAZ1 Gene

Three all-in-one CRISPR/Cas9 lentiviral particles with single guide RNAs (sgRNAs) targeting the second exonic region of the *OAZ1 g*ene were designed and purified by Sigma Aldrich. All-in-one particles encode the sgRNA, Cas9 endonuclease, puromycin resistance, and green fluorescence protein (GFP), driven by a U6 promoter. The sequence of the target regions in the three guide RNAs are as follows:

taacccgggtccggggcctcgg-Sigma Aldrich cat# HS0000288774 (774)

gatcggctgaatgtaacagagg-Sigma Aldrich cat# HS0000288775 (775)

agacgccaaacgcattaactgg-Sigma Aldrich cat# HS0000288778 (778)

### 2.2. Transduction of Human Embryonic Kidney 293 Luciferase Expressing Cells with Lentiviral Particles and Isolation of Single Colonies

Ninety-six well plates (Corning cat #356717, Kennebunk, ME, USA) were coated with 1% Matrigel (Corning cat# 354248) and seeded with 75,000 CMV-Luc2-Hygro HEK293 cells per well in 30 µL of culture media. Plates were incubated overnight in a humidified incubator at 37 °C, 5% CO_2_. Lentiviral particles, three *OAZ1* targeting lentiviral constructs, and CRISPR-Lenti non-targeting control transduction particles (Sigma-Aldrich # CRISPR12V, St Louis, MO, USA) were added the following day at a previously optimized multiplicity of infection (MOI) of 5, in 50 µL media and incubated overnight as above. The media were replaced for an additional overnight incubation. The media were replaced with selection media, containing 1 µg/mL puromycin, on day three post-transduction. The selection media were replaced every other day until confluence was achieved. Limiting dilutions were carried out from each well, as previously described [19,20]**,** in 96-well plates. The wells were scored for the presence of GFP expressing single colonies over a period of two weeks. The wells containing single colonies were propagated and sub-cultured into larger vessels, until enough cells were available for assays (confluent T-25 flask).

### 2.3. Luciferase Assay for Selection of Highly Expressing Clones

Cell viability and luciferase activity were determined using the CellTiter-Glo luminescent cell viability assay and the One-Glo luciferase assay system (Promega cat# G7570 and cat# E6110, Madison, WI, USA, respectively), following manufacturer’s protocol. Briefly, cells at a confluence of 80–90% in a 96-well plate were re-suspended in 100 µL media and transferred to a white opaque 96-well plate (Greiner Bio-one, cat# 655088, Frickenhausen, Germany) in quadruplets. Then, 100 µL of CellTiter-Glo reagent was added to two out of the four quads, mixed for 2 min on a shaker, and allowed to sit at room temperature for 10 min. At approximately 6 min into the 10-min incubation, 100 µL of One-Glo reagent was added to the remaining two wells, mixed briefly, and kept at room temperature until the end of the 10-min incubation period. The luminescence was read using the SpectraMax^®^ microplate reader (Molecular devices, Wals, Austria) at an integration time of 250 ms.

### 2.4. Sequencing of the Ornithine Decarboxylase Antizyme 1 Gene in Parental and Mutant Strains

Total genomic DNA was isolated using the DNeasy kit (Qiagen, cat#69506, Hilden, Germany), following the manufacturers protocol. The genomic region flanking the CRISPR target site for the *OAZ1* gene was polymerase chain reaction (PCR) amplified, using the Phusion^®^ High-Fidelity PCR Master Mix with HF Buffer (NEB, cat# MO531S, Ipswitch, MA, USA), and the following primers: forward primer—cagcagcagtgagagttcca; reverse primer—gcttttggagagcaatggag. Amplicons were gel-extracted using the QIAquick^®^ Gel Extraction Kit (QIAGEN, Ref# 28704, Hilden, Germany), and sequenced using capillary DNA sequencing. Sequences were aligned with the parental sequence using the Clustal Omega (European Bioinformatics Institute, Cambridge, UK) alignment program.

### 2.5. Growth Characterization Determination

Seven T-25 flasks per cell line were each inoculated with 7 × 10^5^ cells in 5 mL culture media and incubated, as described above. The cells were enumerated each day for seven days, using the CEDEX HiREs cell analyzer/counter (Roche # 7766, Basel, Switzerland), and the levels of glucose and lactate were quantified using the YSI 2900 bio-analyzer (Yellow Springs Instrument Co., Yellow Springs, OH, USA).

### 2.6. Antizyme 1 and Luciferase Western Blot

Parental and CRISPR-treated cells in confluent six-well plates were lysed using radioimmunoprecipitation assay (RIPA) cell lysis buffer (Thermo Scientific, # 89900, Rockford, IL, USA) supplemented with 1× protease and phosphatase inhibitor (Thermo Scientific #1861281, Rockford, IL, USA), and total protein was quantified at A280 using the Nanodrop One^c^ (Thermo Scientific, Rockford, IL, USA). Cell lysate samples containing equal amounts of protein (340 µg) and recombinant firefly luciferase protein (abcam # ab100961, Cambridge, MA, USA) were electrophoresed on a 4–12% bis-tris gel (ThermoFisher, # NP0322BOX, Rockford, IL, USA). Separated proteins were transferred onto a nitrocellulose membrane and antizyme 1 (AZ1) or luciferase was detected using rabbit anti-AZ1 polyclonal antibodies targeting the *N*-terminal region of the AZ1 protein (Sigma-Aldrich, cat# SAB1307119, St Louis, MO, USA )/HRP conjugated anti-rabbit secondary antibodies (ThermoFisher Scientific # 65–6120, Rockford, IL, USA); or mouse anti-luciferase polyclonal antibodies (ThermoFisher, # PA1–179, Rockford, IL, USA)/HRP conjugated goat anti-mouse antibodies (KPL # 474–1806, Gaithersburg, MD, USA). Mouse anti-β-actin monoclonal antibodies (Sigma # A2228) and HRP conjugated goat anti-mouse antibodies (KPL # 474–1806, Gaithersburg, MD, USA) were used to detect β-actin in identical samples. The signal was developed using the SuperSignal^®^ west Pico Chemiluminescent substrate (ThermoFisher, # OD187429, Rockford, IL, USA), and visualized in a LAS-4000 Mini Luminescent Image Analyzer (GE healthcare, # 28955810, Marlborough, MA, USA). The intensity of the Western blot bands were determined using ImageJ software and the luciferase/actin ratio, and fold increase was reported.

### 2.7. Quantitative PCR Analysis

Real time quantitative PCR was done using the SYBR GREEN protocol. Total RNA was extracted from *OAZ1* knockout and parental cells using the RNeasy kit (QIAGEN, cat# 74101, Hilden, Germany). First strand cDNA was synthesized from total RNA using the Maxima First strand cDNA synthesis kit for real time quantitative PCR (RT-qPCR) (Thermo Scientific, # K1642, Vilnius Lithuania). Then, 20 ng of cDNA was amplified in a qPCR using the following primers, *OAZ1*: forward primer—GGAACCGTAGACTCGCTCAT, reverse primer—TCGGAGTGAGCGTTTATTTG; and Luc: forward primer—GTGGTGTGCA GCGAGAATAG, reverse primer—CGCTCGTTGTAGATGTCGTTAG.

Threshold and threshold cycle (Ct) values were determined automatically by the RQ Manager^TM^ Software (Applied Biosystems, Foster City, CA, USA) using default parameters. The comparative cycle threshold (2^−ΔΔ*C*t^ method) was used to analyze the expression levels of genes examined in this study. The abundance of each gene transcript was normalized by glyceraldehyde phosphate dehydrogenase (*GAPDH*) or β Actin (*ACTB*) gene expression levels and expressed in arbitrary units (AU). The relative quantization of gene expression was performed in triplicates for each sample.

### 2.8. Transfection of Cells with Secreted Alkaline Phosphatase Plasmid and Quantification of SEAP Activity

Twenty-four well plates were seeded with 200,000 cells per well in culture media one day prior to transfection. The following day, cells were transfected with 500 ng/well of the pSELECT-zeo-SEAP plasmid, encoding the embryonic secreted alkaline phosphatase (SEAP) driven by a EF-1α/HTLV composite promoter (InvivoGen # psetz-seap, San Diego, CA, USA), using the Lipofectamine^®^ 2000 transfection reagent (Invitrogen # 11668-019, Carlsbad, CA, USA), and following the manufacturer’s protocol. After two days, cell culture media were replaced with selection media (culture media supplemented with zeocin 200 µg/mL), and samples were collected for analysis on day three and day five. Alkaline phosphatase activity in the culture supernatant was quantified using the Quanti-Blue colorimetric assay (InvivoGen #rep-qb1, San Diego, CA, USA), cells were enumerated as described above, and total and specific SEAP activity was determined.

### 2.9. Polyamine Quantification

Five million cells per strain were washed twice in phosphate buffered saline (PBS) and pelleted at 1200 rpm for 5 min. The cells were disrupted with 2.3 mm zirconium beads in a mixture of methanol/water (50:50; *v*/*v*) acidified with 0.1% formic acid. Polyamines in cell extract were quantified using Ultra-High Performance Liquid Chromatography (UHPLC) analysis (Agilent 1290, Agilent Technologies, Santa Clara CA, USA), coupled to hybrid triple quadrupole/ion trap mass spectrometer (QTRAP 5500 from AB Sciex, Vaughan, ON, Canada), on Zorbax Eclipse Plus C18, rapid resolution high density (RRHD) column (2.1 × 50 mm 1.8 micron, Agilent Technologies). The chromatography was performed using ultrapure water containing 0.1% formic acid with 1 mM perfluoro heptanoic acid as solvent B and 0.1% formic acid plus 1 mM perfluoro heptanoic acid in 100% methanol as solvent A. The Liquid chromatograph tandem mass-spectrometry (LC-MS/MS) was run for 8.0 min with a flow rate of 300 μL/min. The gradient elution was performed at 50% solvent A for 0.00–4.50 min, for 4.50–5.25 min 100%, 5.25–6.75 min 50%, and 6.75–8.00 min 50%. The mass spectra were acquired using a Turbo Spray Ionization of 2500 V in positive ion mode, and multiple reaction monitoring (MRM). The curtain gas (nitrogen), CAD (collision activated dissociation), nebulizing, and heating gas were set to 40 psi, medium, 50 psi, and 60 psi, respectively. The temperature of the source was fixed at 500 °C. The mass spectrometer was set to have a dwell time of 50 ms. LC-MS/MS data were processed using Analyst 1.6.1 software (AB Sciex, Vaughan, ON, Canada). The results were compared to internal standard of 1,6-hexanediamine and standard curves for putrescine, spermidine, and spermine protein quantification were carried out in parallel using the modified Lowry assay.

One batch of three samples (5 × 10^5^ cells) of each cell line, prepared from the same passage, was analyzed. The polyamine content and protein concentration was determined for each sample.

### 2.10. Statistical Analysis

Mean values and standard deviation or standard error were calculated using standard methods. *p*-values were determined using the Chi-square test.

## 3. Results

### 3.1. Creating HEK293 OAZ1-Deficient Cell Line

HEK293 luciferase expressing cells were treated with three different lentiviral CRISPR constructs, targeting the coding region of the *OAZ1*, in order to knock out the gene. From the three transduced pools, eight single-cell derived clones were isolated using limiting dilutions. The activity of the constitutively expressed luciferase was measured in the isolated clones and in the parental cell line (Table 1). Among the isolated clones, 775–1 and 775–3 showed 7-fold and 2.8-fold higher specific luminescence, respectively, when compared with the parental cell line, and were selected for further investigation.

**Figure 1:**
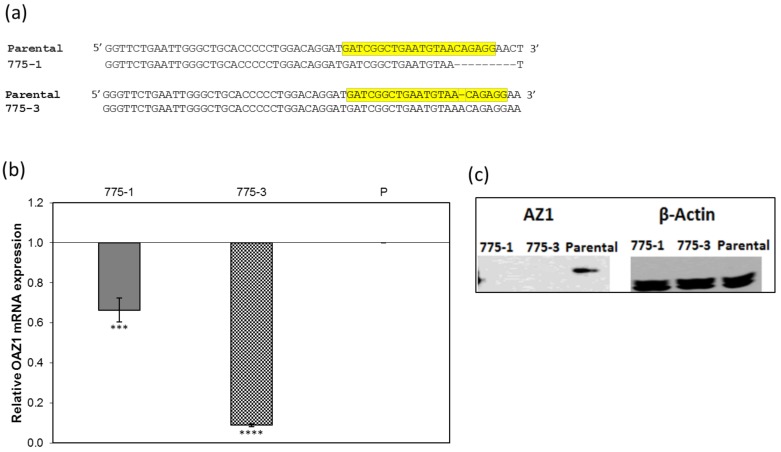
Clustered regularly interspaced short palindromic repeats (CRISPR)-mediated disruption of the ornithine decarboxylase antizyme (*OAZ1*) gene in human embryonic kidney 293 (HEK293)-luc cells and the generation of *OAZ1* knockout cell lines. (**a**) Clustal Omega DNA sequence alignment of the CRISPR *OAZ1* guide RNA (gRNA) target and surrounding regions, showing mutations in the *OAZ1* gene in the CRISPR-treated cell lines, 775–1 and 775–3. Highlighted region is the gRNA target sequence; (**b**) Real time quantitative polymerase chain reaction (RT-qPCR) of reverse-transcribed total cellular mRNA, using primers targeting *OAZ1* cDNA, confirms lower transcriptional levels of *OAZ1* mRNA in the CRISPR-treated cell lines, 775–1 and 775–3, of 0.7-fold (****, *p*-value < 0.00001) and 0.3-fold (***, *p*-value < 0.001) respectively, compared with the parental cell line. β-actin mRNA levels were used as endogenous controls. The assay was carried out in triplicates, and the results represent the average of three independent assays, with error bars representing the standard deviation; (**c**) Western blot analysis confirms *OAZ1* knockout in the 775–1 and 775–3 cell lines. Polyclonal antibodies against the N-terminal of antizyme 1 (AZ1), were used to detect the protein in 340 µg of electrophoresed whole cell lysates after transfer onto a nitrocellulose membrane, while monoclonal antibodies against the loading control β-actin were used to detect the protein in identical samples.

The CRISPR driven disruption of *OAZ1* in the selected cell lines was confirmed by alignment of the CRIPSR treated sequences with the parental sequence. The results shown in Figure 1a revealed the presence of a consecutive nine-nucleotide deletion in the 775–1 *OAZ1* and a single base insertion in the 775–3 *OAZ1*, both of which were confirmed, using PCR, to be bi-allelic (results not shown). Transcriptional and translational analyses determined them to be nonsense mutations, with predicted truncated AZ1 protein of 103 amino acids (775–1) and 128 amino acids (775–3), compared with the 228-full-length protein (Supplemental Figure 1). Consistent with the above findings, RT-qPCR quantification of *OAZ*1 mRNA revealed lower levels of *OAZ*1 mRNA in both knockout cell lines. The 775–1 cell line contained 70% *OAZ1* mRNA, while 775–3 contained about 30% of the *OAZ*1 mRNA (Figure 1b). These findings were confirmed by Western blot, in which AZ1 antibodies did not detect the protein in cell lysates of 775–1 and 773–1 (Figure 1c). The results show that the *OAZ*1 gene was functionally knocked out in both the 775–1 and 775–3 cell lines.

### 3.2. Growth Properties and Metabolic Activity of the OAZ1 Knockout Cell Lines

**Figure 2:**
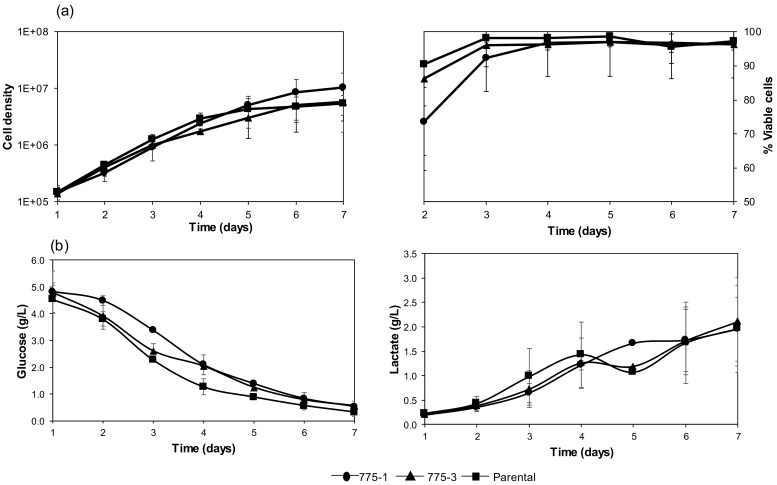
Seven-day growth characterization of the *OAZ1-* cell lines, 775–1 and 775–3, and the parental cell line. (**a**) Proliferation and viability of the *OAZ1*- cell lines 775–1 (●), 775–3 (▲), and the parental (■) cell line. Cells were plated at a density of 7 × 10^5^ cells in T-25 flasks and enumerated using trypan blue exclusion over a seven-day period; and (**b**) glucose and lactate concentration in the culture media quantified using a bioanalyzer over the same seven-day period. Data from two independent growth studies are shown, with error bars representing the standard deviation.

Seven-day growth kinetics and metabolic activity of the 775–1, 775–3 clones, and parental cell lines are shown in Figure 2. Growth rate and viability are shown in Figure 2a, with all cell lines achieving confluency on day six, and exhibiting similar doubling times of approximately 24 h. The *OAZ1* knockout cell lines showed similar viabilities to the parental cell line. The results show that the *OAZ1* inactivation and the increased burden of improved protein expression had minimal effects on the growth and viability of the host cell. The glucose consumption and the lactate production of the knockout and parental cell line were also similar, in accordance with the growth data (Figure 2b).

### 3.3. Transcription Expression and Luciferase Activity in the OAZ1 Knockout Cell Line

**Figure 3:**
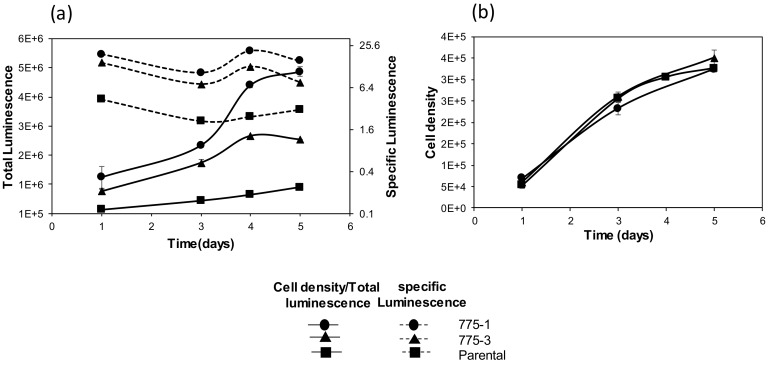
Time course growth and luciferase activity from parental and *OAZ1* deleted cells. Cells were seeded in duplicates, in 12-well plates at 1 × 10^5^ cells per well. (**a**) Total and specific luciferase activity quantified for a five-day period; and (**b**) cell density determined by luminescence based assay for the same five-day period. Graphs represent one of three independent studies and error bars represent standard deviation of duplicate measurements.

**Figure 4:**
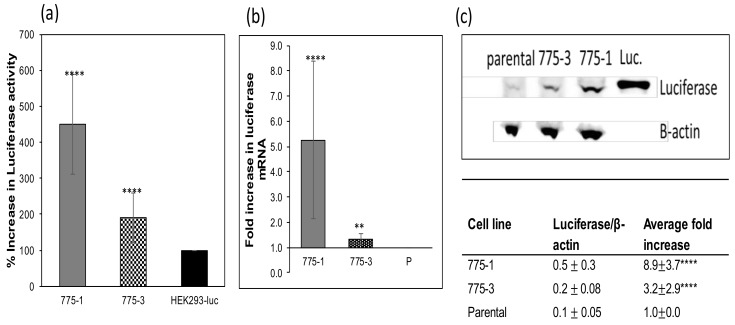
Luciferase activity, transcription, and expression. (a) Luciferase was quantified and reported as described above. The results represent the average of at least five independent assays, each carried out in duplicates, and error bars represent the standard deviation (****, *p*-value < 0.0001). (b) RT-qPCR of reverse-transcribed total cellular mRNA using primers targeting luciferase cDNA confirms higher transcriptional levels of luciferase mRNA in the *OAZ1-* cell lines of approximately 500% for 775–1 (****, *p*-value < 0.0001) and approximately 150% for 775–3 (**, *p*-value < 0.05), compared to the parental cell line. The assay was carried out in triplicates, and the results represent the average of three independent assays, with error bars representing the standard deviation. (c) Western blot analysis of 340 µg of whole cell lysates and 100 ng of recombinant luciferase protein for luciferase and β-actin expression, using anti-luciferase and anti-β-actin primary antibodies showing higher levels of intracellular luciferase protein in the knockout cell lines, 775–1 and 775–3, than in the parental cell line. ImageJ program was used to quantify the band intensities. β-actin-normalized luciferase band intensities were used to determine the fold increase in luciferase expression in the *OAZ1-* cell lines, 775–1 (8.9-fold; ****, *p*-value < 0.00001) and 775–3 (3.2-fold; ****, *p*-value < 0.00001), over the parental cell line. The results represent the average and standard deviation of three independent experiments, with a representative blot shown.

Comparison of total and specific luciferase activity from the *OAZ1* knockout and the parental cells are shown in Figure 3a,b. Specific luciferase activity was not affected by the cell growth and, as expected, there was correlation between total activity and cell density. Quantification of total luciferase activity, messenger RNA level by RT-qPCR, and luciferase protein by immunoblot in samples at equivalent growth stages are shown in Figure 4. Higher specific and total luciferase activity observed in the *OAZ1* knockout cell lines (Figure 4a) were the result of an increase in both mRNA (Figure 4b) and protein expression (Figure 4c).

### 3.4. Expression of Secreted Alkaline Phosphatase in the OAZ1-Deficient HEK 293 Cell Line

**Figure 5:**
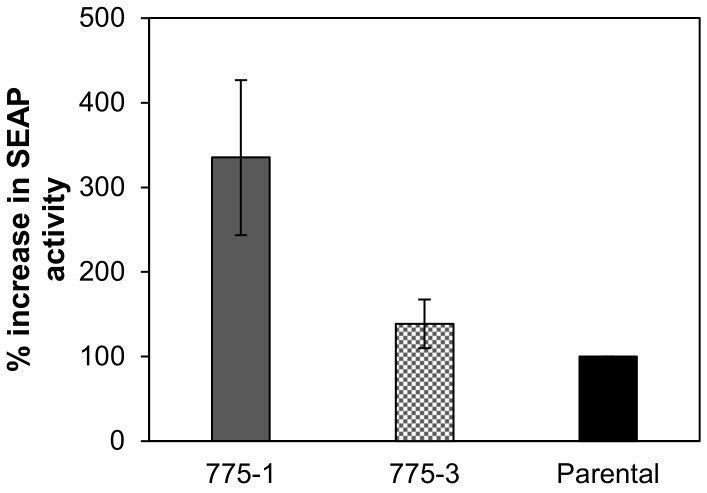
Activity of transiently transfected secreted alkaline phosphatase in *OAZ1-* and parental cell lines. Alkaline phosphatase (AP) activity assayed on day three and day five in the cell culture supernatant of 775–1, 775–3, and parental cells transfected with a plasmid encoding human fetal secreted AP (SEAP). SEAP activity is reported as the percent increase in specific activity (activity per cell), in the *OAZ1* cell lines of (335 ± 92)% for 775–1 and (138 ± 29)% for 775–3, relative to the that of the parental cell line. The results represent the average of two independent transfections, with a total of four biological replicates. Error bars represent the standard deviation.

To evaluate the protein expression capability of the HEK *OAZ1*-deficient cell line, the cells were transiently transfected with plasmid encoding SEAP. Alkaline phosphatase activity was quantified in cell culture supernatant on the third and fifth day post-transfection, together with viable cell density. Consistent with the activity of the constitutive luciferase, the alkaline phosphatase activity was higher in the knockout cell lines, with a 3.5-fold and 1.5-fold increase in the 775–1 and 775–3 cell lines, respectively (Figure 5).

### 3.5. Polyamine Concentration in the Parental and the OAZ1-Defficint Cell Line

The known function of antizyme 1 is negative regulation of intracellular polyamine concentration. The effect of the knockout of *OAZ1* on cellular polyamine levels was thus investigated. Cells grown to confluence were enumerated, and the total amount of polyamine in the equivalent numbers of cells was quantified using UHPLC. Three batches of cells from each cell line were analyzed. The picomole amount of putrescine, spermine, and spermidine per microgram protein are summarized in Table 2. Compared with the parental cells, higher concentration of intracellular polyamines was detected in the *OAZ1* knockout cell lines; it was 2-fold higher in the 775–1 and 3.5-fold higher in the 775–3, with the most significant difference observed in the level of putrescine, where it was 10-fold higher in 775–1, and 88-fold higher in the 775–3 mutants.

## 4. Discussion

Mammalian cell lines are the producers of choice for many recombinant therapeutic proteins. Among the currently utilized producing cell lines, human cell lines are becoming more relevant because of their innate ability to perform the correct post-translation modifications needed for stable and functional proteins [21,22]. However, compared with their non-human counterparts, their productivity is relatively low, necessitating the development of efficient cell lines using genetic engineering, and the improvement of growth and expression strategies.

In an effort to identify genes potentially affecting recombinant protein expression, a high throughput screen evaluating the effect of siRNA gene knock down on luciferase expression from HEK293 cells was conducted [17]. From a total of approximately 20,000 evaluated genes, *OAZ1* was identified as a promising candidate, whose deletion could improve protein expression, and was confirmed by transient transfection of siRNA against *OAZ1*.

The work presented here describes the creation of the HEK293 cell line with *OAZ1* deletion using CRISPR genome editing. Two single clone-derived HEK293 luciferase expressing cell lines deficient in the *OAZ1* gene product, antizyme 1, were created by targeting the second exon of *OAZ1* for disruption. The premise of targeting exon 2 (*OAZ1* has six exons) was to cause disruptions early in the sequence, which would likely result in a truncated or functionally inactive protein, because the ornithine decarboxylase (ODC) binding site is in the internal (122–144) and C-terminal (211–218) portions of the protein [23]. As a result, two mutants with the predicated truncated antizyme 1 sequences of 103 and 128 amino acids, structurally incapable of binding ODC, were selected.

Compared with the parental cell line, both engineered cell lines demonstrated higher expression of luciferase, a stably transfected cytoplasmic protein, and alkaline phosphatase, a transiently transfected secreted protein. The growth kinetics and the metabolic activity of the mutant cell lines were comparable to the parental cell line and, as expected, their intracellular polyamine content was higher.

The improved expression of recombinant proteins exhibited by these *OAZ1* knockout cell lines can be attributed to the observed increase in the level of polyamines, which is likely the result of a missing functional *OAZ1* gene. Antizyme 1, the protein product of *OAZ1,* is known to regulate polyamine production by inhibiting ODC [15,16]. Inhibiting AZ1 has been shown to cause an increase in intracellular polyamines levels, particularly putrescine [24]. The disproportionate effect of *OAZ1* knockout on the different polyamines can be attributed to the *OAZ1*-independent catabolism of spermine and spermidine, but not of putrescine [25]. Adding exogenous polyamines to cell culture of HEK293 has also been shown to enhance recombinant protein expression in a concentration dependent manner [17].

Improved expression of recombinant proteins from the *OAZ1* deficient cells was observed both in cells stably expressing luciferase, and from the same cells transiently transfected with secreted alkaline phosphatase, although to a lesser extent. This suggests that the presented approach for improved expression can be applied to different recombinant proteins, and perhaps different cell lines, although likely with different levels of enhancement depending on the properties of the expressed proteins. The increase in luciferase activity of the knockout cell lines is accompanied by an increase in both mRNA transcription and protein expression. By comparing the increase of mRNA and protein, it was concluded that the increase in expression was engendered at the transcriptional level, with little to no post-transcriptional effects. This observation is different from the initial results obtained from the siRNA silencing of the *OAZ1* gene, in which there was no increase in luciferase mRNA [17].

Improved protein expression is often accompanied by undesirable side effects, such as growth and metabolic disadvantages [26,27], caused by increased metabolic load on the cells. This was not observed in the *OAZ1* deficient or knockout cell lines, where no significant differences in growth and nutrient utilization were observed. These observations can be explained by the known role of *OAZ1* as a negative regulator of cell growth [28], meaning its absence might offset the possible negative effects on cell growth engendered by improved protein expression. It is also possible that the effect of metabolic load is cell line dependent [29]. A peculiar phenomenon is that although both knockout cell lines have higher intracellular polyamine concentrations than the parental cell line, the observed relationship between polyamine concentration and recombinant protein expression is not proportional. The lower producing cell line, 775–3, has at least a two-fold higher polyamine concentration than the higher producing cell line, 775–1. This discrepancy can be explained by possible toxic effects of elevated intracellular polyamine levels, which were not high enough to cause significant cytotoxicity, on the rate of protein synthesis [30]. The previous report by Xiao et al. also showed that improved expression by addition of external polyamines is concentration-dependent up to an optimal concentration, above which higher concentrations have negative effects on the expression [17].

The work presented here was done in anchorage dependent cells growing in serum supplemented media, which are, therefore, limited in their capacity to scale up. The next step would be to investigate the effect of the *OAZ1* deletion in cells growing in suspension, which are more relevant to large-scale recombinant protein production.

## Figures and Tables

**Table 1:** Luciferase enzymatic activity and fold increase in the luciferase activity of isolated single colonies, parental cell line, and negative control cell line. Luciferase activity and cell density were quantified in a luminescence-based assay. Specific luminescence represents luminescence per cell. Parental: human embryonic kidney 293 (HEK293) cell line constitutively expressing luciferase; 774–1, 774–2, 774–3, 775–1, 775–2, 775–3, 778–1: isolated single colonies. Specific luminescence values represent the average of duplicate samples and the standard deviation. RLU = Relative luminescence units.

Single Colony-Derived Clone	Specific Luminescence (RLU/Cell)	Fold Increase in Specific Luciferase Activity
774–1	3.0 ± 1.0	0.6 ± 0.3
774–2	2.2 ± 0.1	0.4 ± 0.0
774–3	6.2 ± 1.0	1.1 ± 0.2
775–1	40.1 ± 2.4	7.0 ± 0.4
775–2	3.2 ± 0.6	0.6 ± 0.2
775–3	16.1 ± 0.7	2.8 ± 0.1
775–4	4.5 ± 3.2	0.8 ± 0.6
778–1	6.4 ± 0.1	1.1 ± 0.0
Parental	5.8 ± 1.9	1.0 ± 0.0
Negative control	4.4 ± 1.2	0.8 ± 0.2

**Table 2:** Intracellular polyamine levels in ornithine decarboxylase antizyme 1 deficient *(OAZ1-)* and parental cell lines. The intracellular levels of the polyamines putrescine, spermine, and spermidine in 5 × 10^5^ pelleted cells were quantified chromatographically, with simultaneous quantification of total protein, using the modified Lowry assay. Polyamine content is reported relative to the total protein content of the sample. The results represent the average of three samples from a single batch of each cell line prepared from the same passage, and the standard deviation.

	Polyamines (pmol/µg Protein)			
Cell Line	Total Polyamine	Putrescine	Spermidine	Spermine
775–1	20.3 ± 2.1 ****	2.5 ± 0.7 ***	12.8 ± 2.4 **	5.0 ± 1.3
775–3	38.1 ± 2.0 ****	21.9 ± 1.3 ***	13.4 ± 0.6 **	2.8 ± 0.4
Parental	11.7 ± 1.3	0.2 ± 0.0	8.2 ± 0.4	3.3 ± 0.9

** *p*-value < 0.05; *** *p*-value < 0.001; **** *p*-value< 0.0001.

## Supplementary Materials

The following are available online at http://www.mdpi.com/2076-3271/6/2/48/s1.
