# Supplementary material for: Knocking out Ornithine Decarboxylase Antizyme 1 (OAZ1) Improves Recombinant Protein Expression in the HEK293 Cell Line
Source: Med Sci (Basel). 2018 Jun 8;6(2):48. doi: 10.3390/medsci6020048 (PMC6024716; doi:10.3390/medsci6020048)
Supplement: Supplementary file 1 [file medsci-06-00048-s001.pptx]

## Slide 1
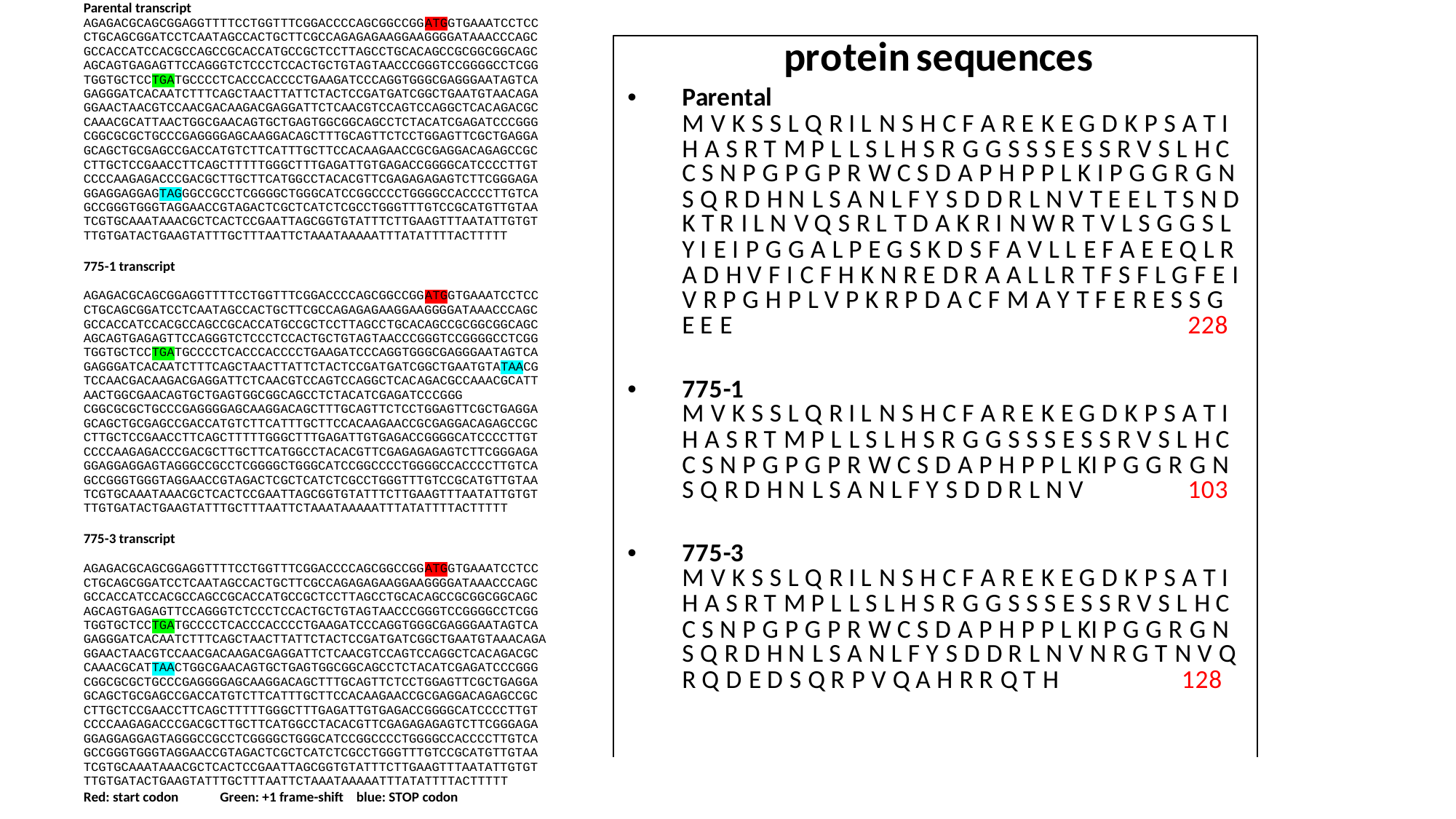

## Slide 2
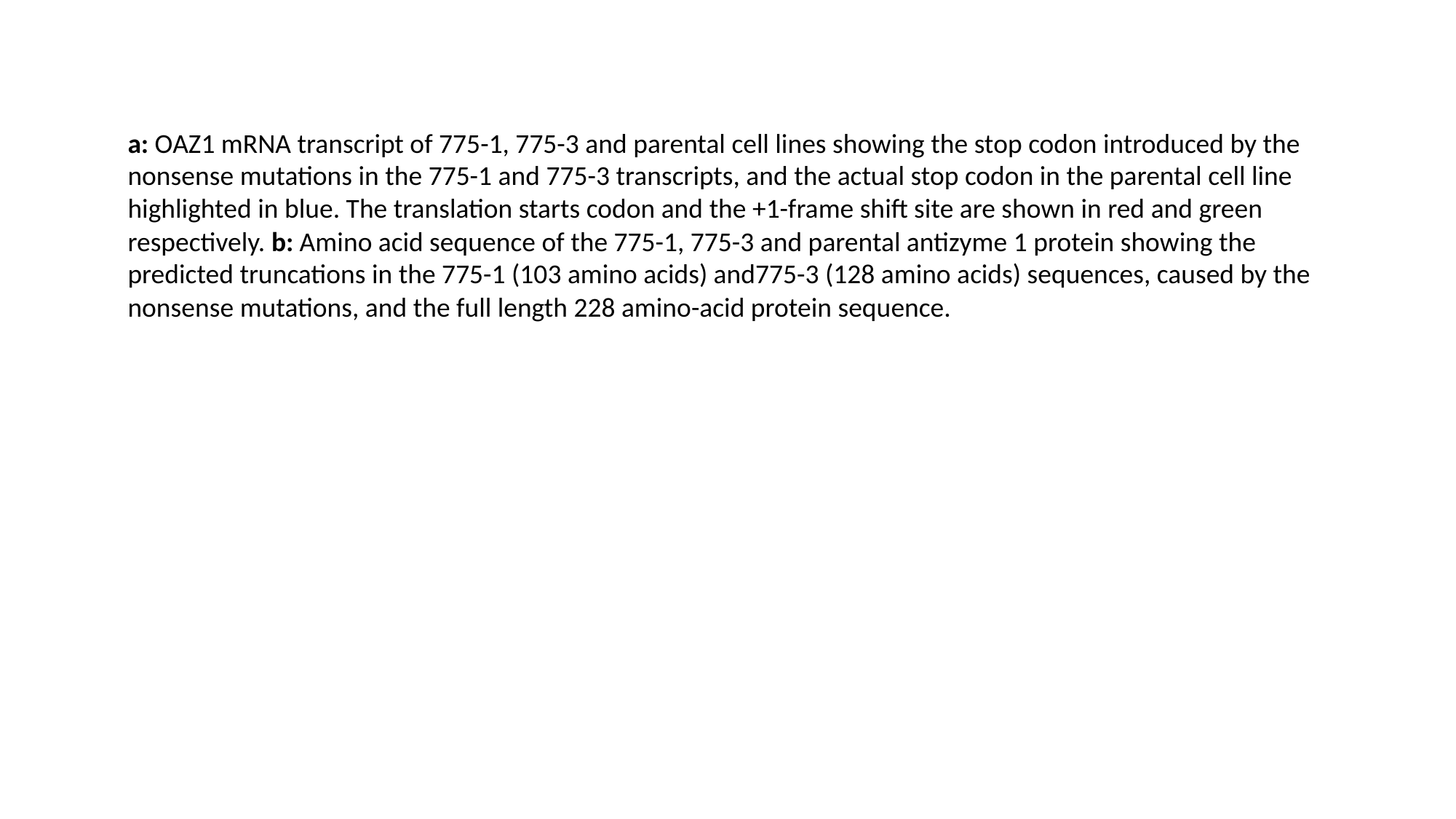

a: OAZ1 mRNA transcript of 775-1, 775-3 and parental cell lines showing the stop codon introduced by the nonsense mutations in the 775-1 and 775-3 transcripts, and the actual stop codon in the parental cell line highlighted in blue. The translation starts codon and the +1-frame shift site are shown in red and green respectively. b: Amino acid sequence of the 775-1, 775-3 and parental antizyme 1 protein showing the predicted truncations in the 775-1 (103 amino acids) and775-3 (128 amino acids) sequences, caused by the nonsense mutations, and the full length 228 amino-acid protein sequence.
